# The risk model construction of the genes regulated by H3K36me3 and H3K79me2 in breast cancer

**DOI:** 10.52601/bpr.2023.220022

**Published:** 2023-02-28

**Authors:** Ling-Yu Wang, Lu-Qiang Zhang, Qian-Zhong Li, Hui Bai

**Affiliations:** 1 Laboratory of Theoretical Biophysics, School of Physical Science and Technology, Inner Mongolia University, Hohhot 010021, China; 2 The State Key Laboratory of Reproductive Regulation and Breeding of Grassland Livestock, Inner Mongolia University, Hohhot 010070, China

**Keywords:** Breast cancer, Gene expression, H3K79me2, H3K36me3, Driver genes

## Abstract

Abnormal histone modifications (HMs) can promote the occurrence of breast cancer. To elucidate the relationship between HMs and gene expression, we analyzed HM binding patterns and calculated their signal changes between breast tumor cells and normal cells. On this basis, the influences of HM signal changes on the expression changes of breast cancer-related genes were estimated by three different methods. The results showed that H3K79me2 and H3K36me3 may contribute more to gene expression changes. Subsequently, 2109 genes with differential H3K79me2 or H3K36me3 levels during cancerogenesis were identified by the Shannon entropy and submitted to perform functional enrichment analyses. Enrichment analyses displayed that these genes were involved in pathways in cancer, human papillomavirus infection, and viral carcinogenesis. Univariate Cox, LASSO, and multivariate Cox regression analyses were then adopted, and nine potential breast cancer-related driver genes were extracted from the genes with differential H3K79me2/H3K36me3 levels in the TCGA cohort. To facilitate the application, the expression levels of nine driver genes were transformed into a risk score model, and its robustness was tested via time-dependent receiver operating characteristic curves in the TCGA dataset and an independent GEO dataset. At last, the distribution levels of H3K79me2 and H3K36me3 in the nine driver genes were reanalyzed in the two cell lines and the regions with significant signal changes were located.

## INTRODUCTION

Breast cancer is the leading cause of cancer-related deaths and poses a serious threat to women’s lives and health. Early-stage breast cancer is a potentially curable disease, and treatments for these patients include radiotherapy, endocrine therapy, chemotherapy, and HER2 molecular-targeted therapy. In contrast to early-stage breast cancer, metastatic breast cancer is considered incurable with currently available therapies (Harbeck and Gnant 2017). In the past decades, although the prognosis of breast cancer has been improved due to advances in early diagnosis and comprehensive screening, lower survival rates suggest that other clinically relevant biomarkers or therapeutic targets still need to be mined.

Studies have reported that epigenetics plays fundamental roles in breast cancer development and progression (Hinshelwood and Clark 2008). Abnormalities in chromatin structure regulated by epigenetic modifications will affect cellular plasticity and facilitate the oncogenic reprogramming of tumor progenitor cells (Pasculli *et al.* 2018). And epigenetic changes can alter cancer cell phenotype and metastatic potential (Jin *et al.* 2020; Nienhuis *et al.* 2015). As a vital research topic of epigenetics, histone modifications (HMs) participate in the development of breast cancer. For example, hypomethylated HMs can activate breast cancer-related fibroblasts (Lee *et al.* 2020), the genome-wide gain of H3K4ac associates with early and advanced breast cancer cell phenotypes, and the gain of H3K4me3 is mainly related to late-stage cancer cells (Messier *et al.* 2016). These findings indicate that HM levels and distributions may also lead to tumorigenesis.

Here, we firstly analyzed the distribution patterns of 11 important histone acetylation and methylation modifications shared by breast normal and tumor cell lines in the ENCODE database. And the influences of HMs on gene expression changes were explored by three strategies. On this basis, the genes with differential H3K79me2 or H3K36me3 levels between the breast tumor and normal cells were extracted. And these genes were submitted to univariate Cox, LASSO and multivariate Cox regression analyses to identify potential breast cancer-related driver genes. The expression levels of these driver genes were then transformed into a breast cancer prognostic risk score model, and its robustness was validated in the TCGA dataset and an independent GEO cohort. At last, the distribution levels of H3K79me2 and H3K36me3 in these driver genes were reanalyzed in breast tumor and normal cell lines and the regions with significant signal changes were located.

## RESULT

### Histone modification signals change significantly in breast cancer

**Figure 1:**
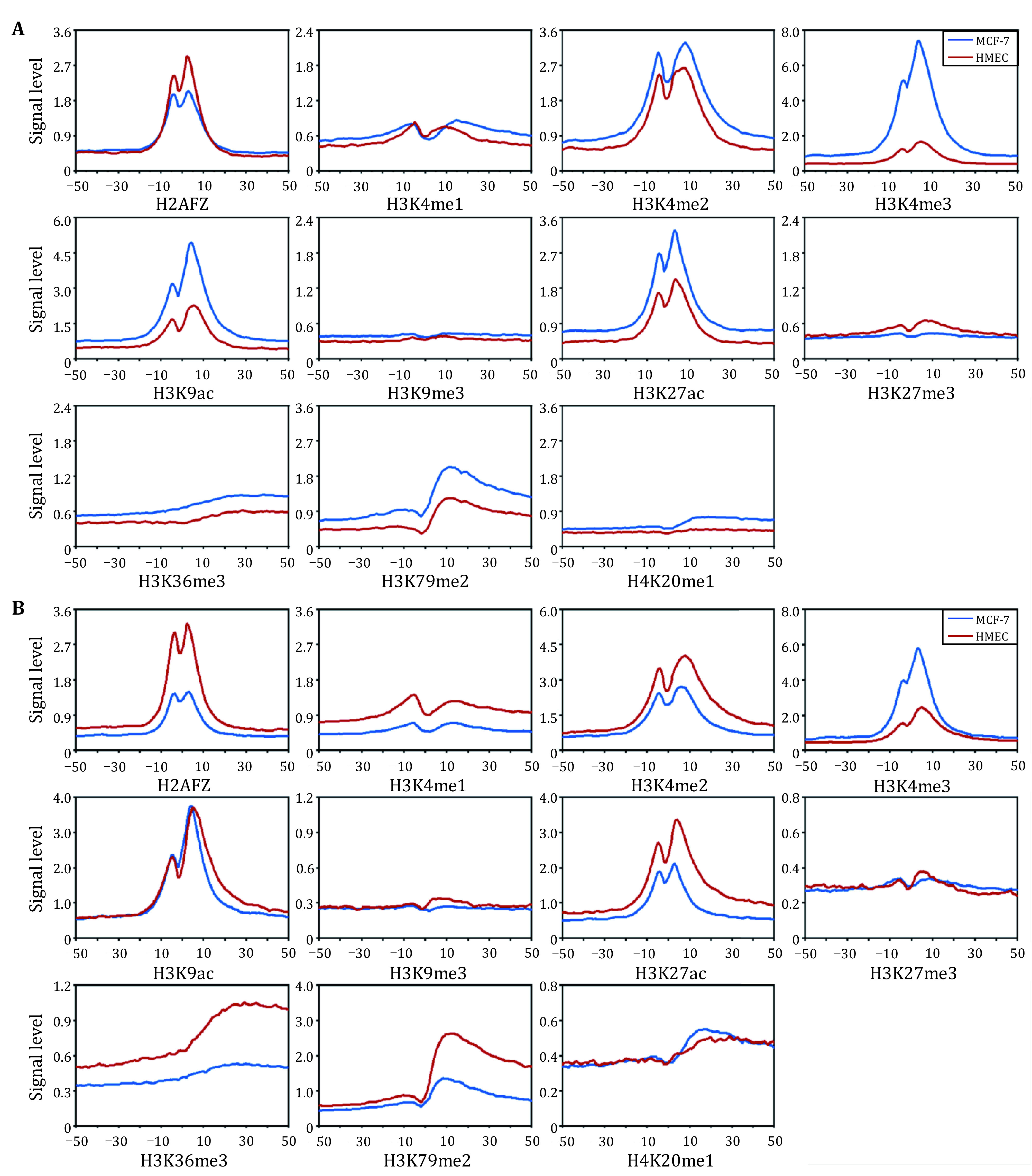
Signal distribution of HMs within the 100 bins of the up- and down-DEGs in the breast tumor and normal cells. **A** Up-regulated genes. **B** Down-regulated genes. MCF-7 and HMEC correspond to breast tumor and normal cells

Recent studies have reported that abnormal HMs can adversely affect the expression of genes in breast cancer (Pasculli *et al.* 2018; Rahman *et al.* 2019). To quantify the dynamic changes of HMs, we firstly identified the differentially expressed genes (DEGs) during tumorigenesis via Eq. 1 and Eq. 2. And the binding signals of 11 HMs across the 100 bins in the up- and down-DEGs within the breast tumor and normal cells were calculated using Eq. 3. Among the up-DEGs (Fig. 1A), some HMs, such as H3K79me2 and H3K36me3, increased obviously in breast tumor cells than that in the breast normal cells, especially in the downstream regions of TSS. However, compared with the signals in normal cells, H3K27me3 decreased significantly in tumor cells, suggesting that H3K27me3 acts as an inhibitory HM, which was consistent with previous research results (Nair *et al.* 2018). Among the down-DEGs (Fig. 1B), almost all HMs including H3K79me2 and H3K36me3 were remarkably decreased in tumor cells.

### H3K79me2 and H3K36me3 have better abilities to discriminate up- from down-regulated genes

**Figure 2:**
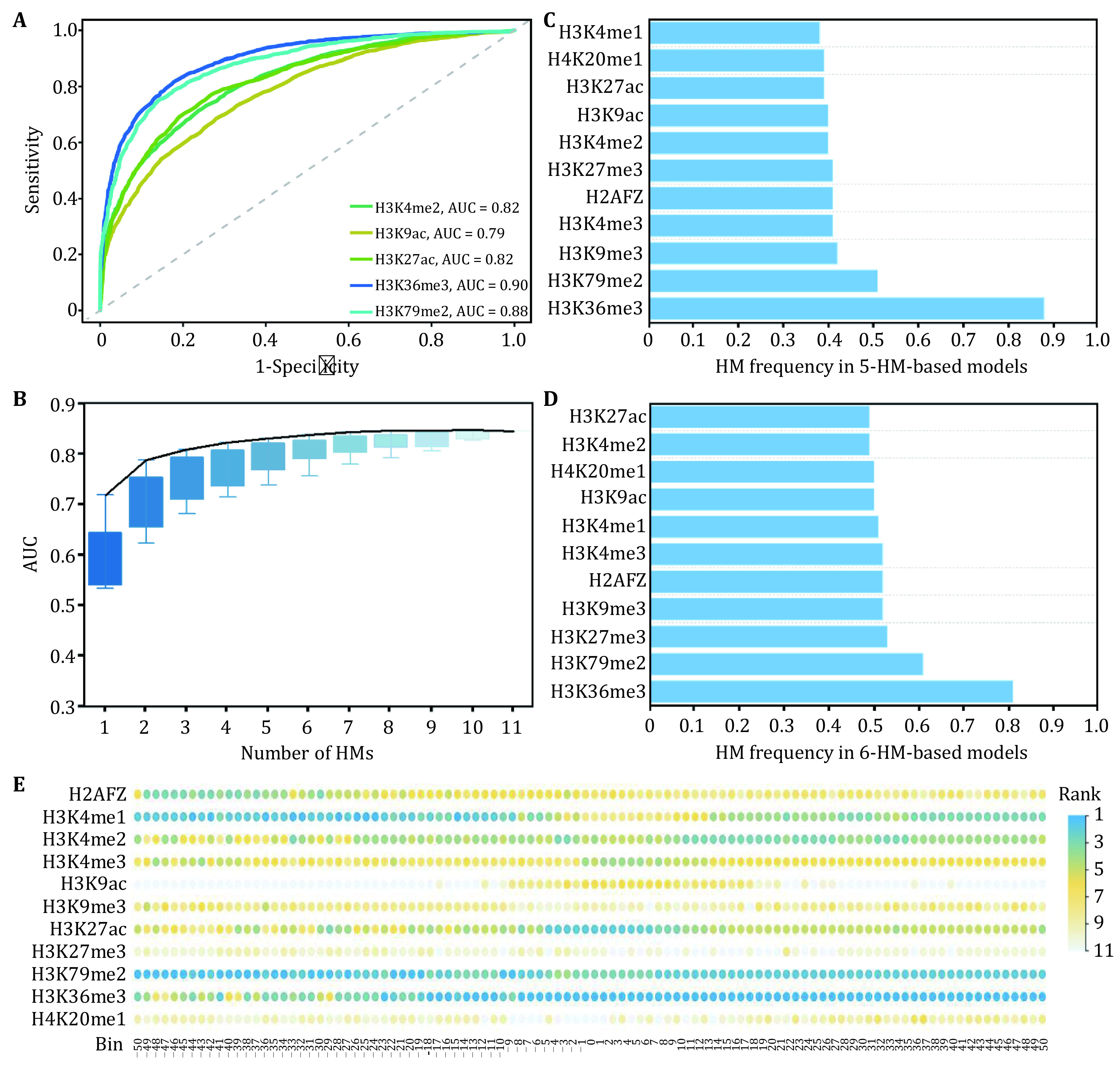
Estimation of the effects of histone modifications on gene expression changes. **A** ROC curves for the five HMs with better predictive abilities. **B** The predictive abilities of the combined models for *n* (*n* = 1, 2, ..., 11) kinds of HMs. HMs’ frequency in the studied 5-HM-based models (**C**) and 6-HM-based models (**D**) whose predictive abilities are greater than 95% of the predictive ability of the 11-HMs-based model. **E** Rank of 11 HMs in the same bin. HMs with lower rank values represent higher IncMSE values and greater contributions to gene expression changes

To estimate the roles of HMs in the regulation of breast cancer-related gene expression, based on the HM signals in the 100 bins, the up-DEGs and down-DEGs were predicted by the random forest (RF) algorithm. Among the 11 HMs, H3K36me3 and H3K79me2 displayed better prediction abilities, and their prediction results were, respectively, AUC = 0.90 and AUC = 0.88. The top five HMs and their predictive results were shown in Fig. 2A.

Among the regulatory mechanisms affecting gene expression, HMs can regulate gene expression in cooperative manners (Audia and Campbell 2016; Morgan and Shilatifard 2020). Therefore, we focused on the impacts of HMs combinations on breast cancer-related gene expression changes. For the 11 HMs, a total of 2047 combinations were obtained by selecting *n* (*n* = 1, 2, ..., 11) kinds of HMs from the 11 HMs. We subsequently calculated the predictive abilities of all combinations to gene expression changes. And the *n*-HMs-based combination with the highest prediction accuracy was connected by a black curve (Fig. 2B). From Fig. 2B, we noticed that the predictive powers increased slightly after using more than five types of HMs. The best five-HMs-based combination (AUC = 0.85) includes H3K4me1, H3K9me3, H3K27me3, H3K36me3 and H3K79me2. And each of the top five 5-HMs-based models contains at least one of H3K36me3 and H3K79me2 (Table 1). We then calculated the frequencies of 11 HMs in the 5-HM-based combinations whose predictive abilities exceeded 95% of the predictive ability of the 11-HMs-based combination. Among the studied 5-HMs-based combinations, H3K36me3 appears in most of the combinations, with a frequency of 87.5%. It is closely followed by H3K79me2, which occurs with a frequency of 50.93% (Fig. 2C). Same analyses were executed for the 6-HMs-based combination, and analogous consequences were found (Fig. 2D). These analyses once again proved the important regulatory roles of H3K79me2 and H3K36me3 in breast cancer.

**Table 1:** The combinations of the top five 5-HMs-based models

AUC	Combination of HMs				
0.853	H3K4me1	H3K9me3	H3K27me3	H3K36me3	H3K79me2
0.850	H3K4me1	H3K4me2	H3K9me3	H3K36me3	H3K79me2
0.850	H3K4me2	H3K27ac	H3K27me3	H3K36me3	H4K20me1
0.849	H3K4me2	H3K4me3	H3K27me3	H3K36me3	H4K20me1
0.848	H3K4me3	H3K9ac	H3K27ac	H3K36me3	H3K79me2

For further validation, the 11 HM signals in the same bin were selected as the information parameters of the RF algorithm to predict the up- and down-DEGs. The results were described in Fig. 2E. As shown, H3K36me3 displayed high predictive abilities in almost all 100 bins, followed by H3K79me2. And H3K79me2 played its more important roles in the downstream regions of the TSS. These results together revealed that H3K79me2 and H3K36me3 maybe two important driving factors in breast cancer development and progression.

### Functional enrichment analyses for genes with differential H3K79me2 or H3K36me3 levels during cancerogenesis

**Figure 3:**
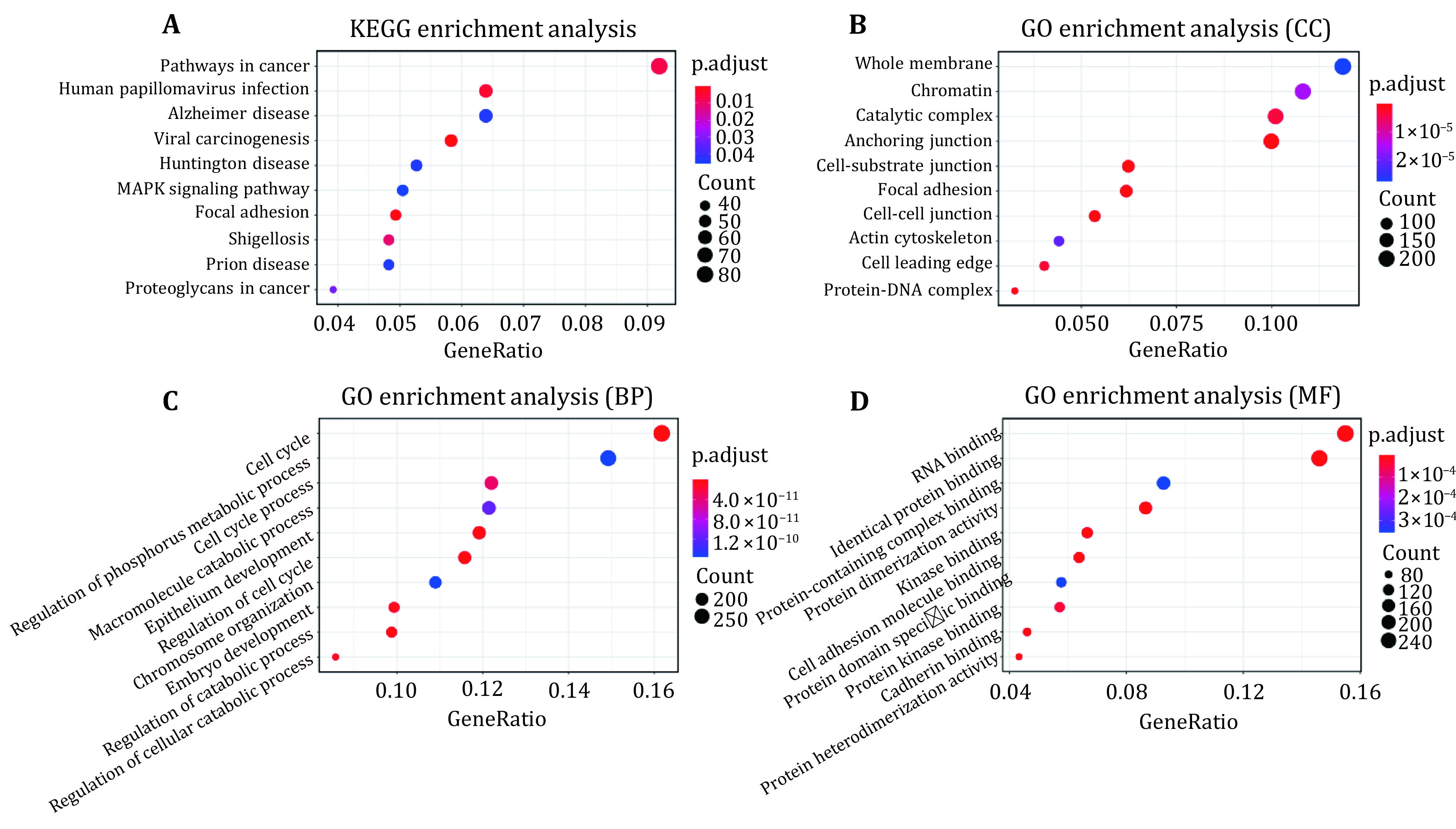
Enrichment analysis for the 2109 candidate genes. **A** KEGG pathway analysis for candidate genes. Significant GO cellular component terms (**B**), GO biological process terms (**C**) and GO molecular function terms (**D**) for candidate genes

To further verify the prognostic values of H3K36me3 and H3K79me2, we identified 2109 genes with differential H3K79me2/H3K36me3 levels between breast cancer and normal cell lines according to Eq. 5. Among these genes, 972 genes display differential H3K79me2 levels and the rest are accompanied by different H3K36me3 levels during cancerogenesis. Then, the Hiplot software was used to perform enrichment analyses for these genes. The top ten significant GO terms and KEGG pathways were listed in Fig. 3. The functional categories and pathways analyses revealed that these genes involved in pathways including pathways in cancer, human papillomavirus infection, viral carcinogenesis, MAPK signaling pathway and proteoglycans in cancer (Santarpia *et al.* 2012; Wagner and Nebreda 2009; Williams and Stoeber 2012). These cancer-related pathways imply that the genes with differential H3K36me3/H3K79me2 levels during cancerogenesis may be implicated in breast cancer development.

### Model construction via driver genes related to H3K36me3 and H3K79me2

To find out significant driver genes associated with the overall survival of breast cancer patients, we first intersected the 2109 differential H3K79me2/H3K36me3 level genes and DEGs. 166 H3K36me3/H3K79me2-related DEGs expressed in more than 90% TCGA samples were submitted to univariate Cox analysis. As a result, 27 genes with *P*-values < 0.05 were recognized as seed genes. Subsequently, 27 seed genes were incorporated into the LASSO regression analysis, and 18 genes with minimum lambda value were retained. Finally, multivariate Cox regression analysis was employed to construct a prognostic risk score model, which included nine of the 18 genes. The results of the multivariate Cox regression analysis were shown in Table 2. The risk score model was as follows, where the $ {{\overline L_{j,t}}} $ represents the average expression level of the *j*-th gene in tumor samples.

**Table 2:** Summary of multivariable Cox regression analysis

Gene	Coef	Hazard ration	Secoef	*Z*-score	*P*-value
ERRFI1	−0.288	0.750	0.095	−3.047	2.313 $ \times\; {{10}}^{{-3}} $
GREB1	−0.139	0.870	0.041	−3.410	6.485 $ \times\; {{10}}^{{-4}} $
RTN4	0.499	1.648	0.174	2.878	4.008 $ \times\; {{10}}^{{-3}} $
PHF7	−0.216	0.805	0.109	−1.979	4.778 $ \times\; {{10}}^{{-2}} $
SPRY4	0.378	1.459	0.102	3.701	2.151 $ \times\; {{10}}^{{-4}} $
CCND2	−0.290	0.748	0.083	−3.502	4.613 $ \times\; {{10}}^{{-4}} $
RPL19	−0.443	0.642	0.120	−3.703	2.133 $ \times\; {{10}}^{{-4}} $
TBX4	−0.103	0.902	0.053	−1.941	5.227 $ \times\; {{10}}^{{-2}} $
CYP24A1	−0.100	0.904	0.038	−2.655	7.919 $ \times\; {{10}}^{{-3}} $

*RS* = −0.288 × $ {\bar L_{ERRFI1,t}} $ − 0.139 × $ {\bar L_{GREB1,t}} $ + 0.499 × $ {\bar L_{RTN4,t}} $ − 0.216 × $ {\bar L_{PHF7,t}} $ + 0.378 × $ {\bar L_{SPRY4,t}} $ − 0.290 × $ {\bar L_{CCND2,t}} $ − 0.443 × $ {\bar L_{RPL19,t}} $ − 0.103 × $ {\bar L_{TBX4,t}} $ − 0.100 × $ {\bar L_{CYP24A1,t}} $ .

**Figure 4:**
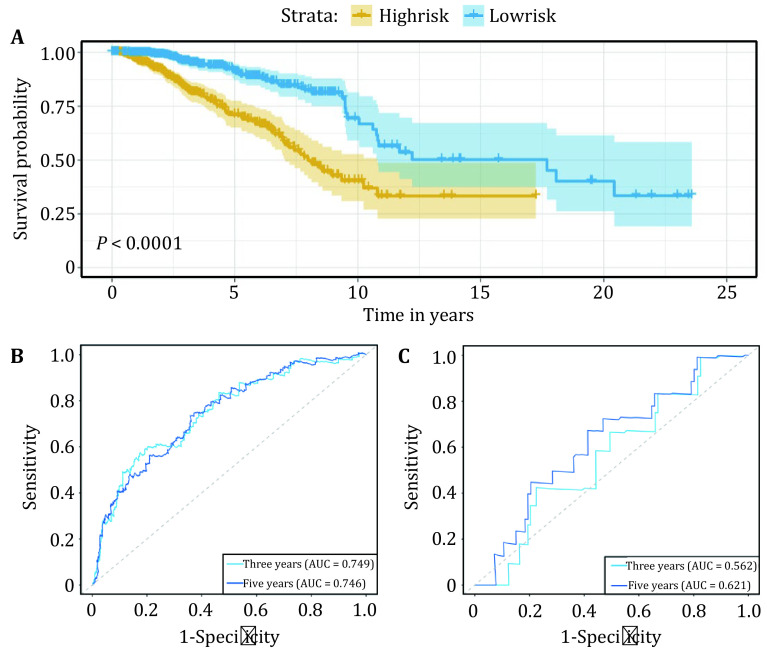
Survival assessment of the risk score model. **A** Kaplan-Meier curves for breast cancer patients in the high- and low-risk groups within the TCGA cohort. Time-dependent ROC curves at three and five years within the TCGA cohort (**B**) and GEO cohort (**C**)

To quantify the prognostic efficiency of the risk score model, breast cancer patients in the TCGA cohort were divided into high-risk and low-risk groups using the median risk score as the cut-off value. And Kaplan-Meier curves were generated to depict the survival difference for patients in the high-/low-risk group. The results showed that patients in the low-risk group displayed higher survival rates than those in the high-risk group (Fig. 4A). The AUCs at three and five years were 0.749 and 0.746, respectively (Fig. 4B).

Moreover, to test the robustness of the risk score model, the model was applied to an independent GEO cohort (GSE162228). For 3-year and 5-year overall survival, the AUCs of the model were 0.562 and 0.621, respectively (Fig. 4C). Overall, these analyses indicated that the model constructed by nine H3K36me3/H3K79me2-related genes can serve as a supplement to existing signatures.

### Signal distribution of H3K36me3 and H3K79me2 in driver genes

**Figure 5:**
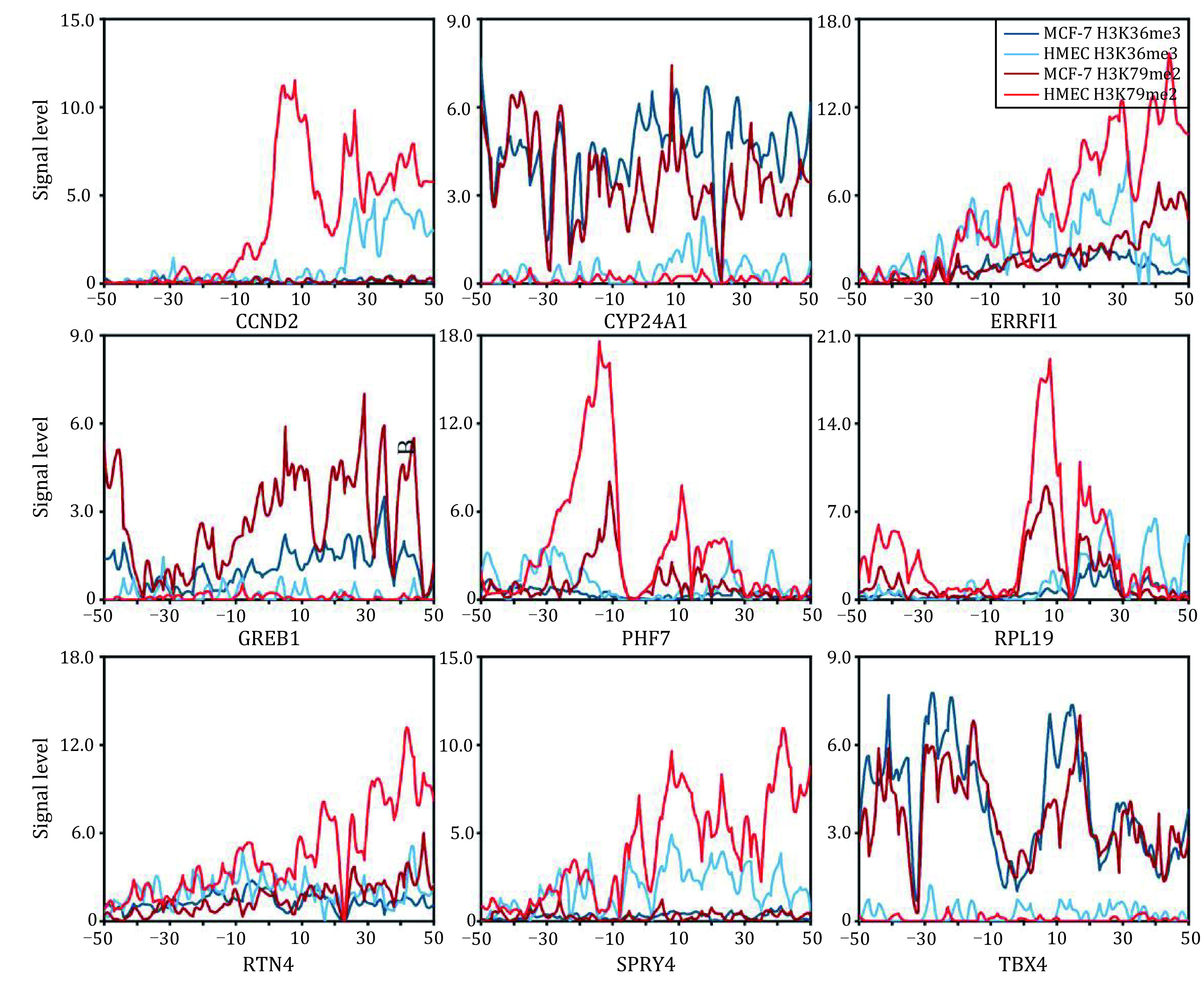
Signal distributions of H3K36me3 and H3K79me2 in the 100 bins of nine genes within breast tumor and normal cells

The prediction of breast cancer-related prognosis revealed that the nine genes are important in the occurrence of breast cancer. To describe the distribution levels of H3K36me3 and H3K79me2 in these nine genes, we re-quantified the binding signals of both HMs across the 100 bins within the breast tumor and normal cells, respectively (Fig. 5). The results displayed that H3K36me3 and H3K79me2 changed significantly in the nine genes during cancerogenesis. For ERRFI1, RTN4, PHF7, SPRY4, CCND2, and RPL19, H3K36me3 and H3K79me2 enriched higher signals in normal cell lines than that in cancer cells, and the signals of H3K79me2 changed more significantly than H3K36me3 during tumorigenesis. While for genes GREB1, TBX4 and CYP24A1, both HMs showed higher signals in cancer cell lines than that in normal cells. Compared with the H3K36me3 signal in RPL19, H3K79me2 enriched more in normal and cancer cell lines.

## DISCUSSION

Understanding the alternations in HMs and the impacts of HM changes on gene expression changes can provide important insights into tumorigenesis mechanisms. In this study, to mine the important HMs in breast cancer, three different analytic methods were presented. The results displayed that the changes of H3K79me2 and H3K36me3 contribute more to gene expression changes, which may be two significant HMs in breast cancer. In fact, our previous findings suggested that H3K79me2 and H3K36me3, two well-studied elongation-related marks, contribute important regulations to gene expression in breast cancer, leukemia, and lung cancer (Jin *et al.* 2020; Zhang *et al.* 2021b, 2022). And gene-body H3K36me3 signals within normal cells negatively regulate gene expression level changes in chronic myelogenous leukemia, and lung/breast cancers regardless of gene expression levels and gene lengths (Zhang *et al.* 2021a). Additionally, emerging lines of evidence suggest that H3K36me3 and H3K79me2 mediate a variety of transcription-related events, such as transcriptional activity regulation, transcription elongation, and RNA m6A methylation (Xiao *et al.* 2021). And they also involve in the regulation of skipping exon processing and T cell immunity (Bian *et al.* 2020; Li *et al.* 2018; Schubeler *et al.* 2004). Our findings, together with previous reports, indicate that H3K79me2 and H3K36me3 may be therapeutic targets for breast cancer. Given that, we immediately execute enrichment analyses for the 2109 differential H3K79me2/H3K36me3 level genes during cancerogenesis. The cancer-related pathways once revealed that the studied genes might become critical therapeutic targets for breast cancer.

Subsequently, we perform univariate Cox, LASSO, and multivariate Cox regression analyses to extract potential breast cancer-related driver genes. Among the identified driver genes, most of them have been demonstrated to be related to breast cancer. ERRFI is generally regarded as a tumor suppressor in human cancers, and microarray analysis has revealed that ERRFI is positively correlated with the survival of breast cancer patients (Amatschek *et al.* 2004; He *et al.* 2021; Mojica *et al.* 2020; Xu *et al.* 2005). GREB1 significantly affects the proliferation of estrogen receptor-α, which is a driving transcription factor in breast cancers and influences drug response (Rae *et al.* 2005). The down-regulation of RTN4 expression leads to a decrease in AKT activation, which is an important event related to breast cancer occurrence and metastasis (Pathak *et al.* 2018). SPRY4 can act as a tumor suppressor or an oncogene depending on human cancer. In breast and prostate cancers, SPRY4 can inhibit cell proliferation and migration (Jing *et al.* 2016; Vanas *et al.* 2014; Wang *et al.* 2006), while in ovarian cancer, SPRY4 promotes ovarian cancer invasion (So *et al.* 2016). RPL19 has been identified as a tumor-specific antigen and a prognostic biomarker in breast cancer (Albanese *et al.* 2018). TBX4 and CYP24A1 are overexpressed in breast cancer and are associated with breast cancer risk (Anderson *et al.* 2011; Kelemen *et al.* 2009). Although there are few reports on the functions of the PHF7 and CCND2 in breast cancer, they are associated with cardiac disease (Eroglu *et al.* 2021), bladder squamous cell carcinoma (Shivakumar *et al.* 2017), and neuroblastoma (Duan *et al.* 2018). In summary, these results indicated that the nine genes maybe the driver genes in breast cancer. In order to promote the clinical application of these driver genes, a risk score model was constructed and the model displayed robust predictive abilities in the TCGA and GEO datasets. Finally, we switched back to the distribution levels of H3K36me3 and H3K79me2 in the nine driver genes. By comparing H3K36me3 and H3K79me2 signals in tumor cells with those in normal cells, we identified the regions where H3K36me3 and H3K79me2 signals were significantly altered.

In this study, although the key HMs, H3K79me2/H3K36me3-related driver genes were investigated, and a prognostic risk model was constructed, there are still some drawbacks. First, the underlying mechanisms of nine driver genes are still unclear in breast cancer. Second, despite the literature validation was performed, additional analyses using experimental validation are still needed in the future.

## MATERIALS AND METHODS

### Data and preprocessing

The human reference genome annotation file (GRCh38) was downloaded from the UCSC database (http://genome.ucsc.edu/). For genes with multiple alternative transcripts, only one of them was randomly selected. Finally, 19307 protein-coding genes on autosomes and X chromosomes were reserved for further analysis.

Genome-wide polyA + RNA-seq and HMs data for HMEC (mammary epithelial cell, normal) and MCF-7 (mammary gland cancer cell, cancer) were downloaded from the ENCODE database (https://www.encodeproject.org/). The HMs shared by tumor and normal cell lines include H2AFZ, H3K4me1, H3K4me2, H3K4me3, H3K9ac, H3K9me3, H3K27ac, H3K27me3, H3K36me3, H3K79me2, H4K20me1. Gene expression levels were quantified by reads per kilobase of exon model per million mapped reads (Mortazavi *et al.* 2008; Su 2021) and calculated by Eq. 1:

1 $ {L_j} = \frac{{{n_j} \times {{10}^9}}}{{N \times {l_j}}}, $

where *L*_*j*_represents the expression level of the *j-*th gene. *n*_*j*_ is the number of polyA + RNA-seq reads falling into the exon region of the *j*-th gene. *N* is the total reads of polyA + RNA-seq, and *l*_*j*_ is the length of the *j*-th gene’s exon.

Raw clinical information and expression data for breast cancer patients were indexed from the TCGA database (https://portal.gdc.cancer.gov/), including 113 normal samples and 1109 tumor samples. Expression levels for genes in the TCGA cohort were standardized by transcripts per million (Wagner *et al.* 2012). Meanwhile, an independent GEO cohort (GSE162228) including clinical and microarray data from 24 normal samples and 109 cancer samples was obtained from the GEO database (https://www.ncbi.nlm.nih.gov/geo/), where the microarray data were normalized via the robust multiarray average algorithm.

### Quantification of differentially expressed genes

The differentially expressed genes (DEGs) between the breast tumor and normal cell lines were identified by Eq. 2. Genes with |log_2_(*FC*)| > 1 and *P*-value < 0.01 were recognized as DEGs. A total of 3821 genes and 3552 genes were, respectively, classified as down-regulated genes (down-DEGs) and up-regulated genes (up-DEGs).

2 $\left\{\begin{aligned} &
{\overline{L}}_{j,t}=\frac{1}{{N}_{t}}{\displaystyle \sum _{n\;=\;1}^{{N}_{t}}{L}_{j,t}};\;{\overline{L}}_{j,n}=\frac{1}{{N}_{n}}{\displaystyle \sum _{n\;=\;1}^{{N}_{n}}{L}_{j,n}}\\& {\mathrm{log}}_{2}(FC)={\rm{log}}_{2}({\overline{L}}_{j,t}/{\overline{L}}_{j,n})\\&
P{\text{-}}value=({\overline{L}}_{j,t}/{\overline{L}}_{j,n})\Bigg/\sqrt{\frac{{S}_{j,t}^{2}}{{N}_{t}} + \frac{{S}_{j,n}^{2}}{{N}_{n}}}
\end{aligned}\right.\;\; , $

where ${\bar L_{j,t}}$ and ${\bar L_{j,n}}$ respectively denote the average expression levels of the *j*-th gene across the $ {N_t} $ tumor samples and $ {N_n} $ normal samples. $ S_{j,t}^2 $ and $ S_{j,n}^2 $ represent the variances of the *j*-th gene expression level changes across the tumor and normal samples.

### Calculation of histone modification binding signals

To calculate the signal distributions of HMs in tumor and normal cell lines, the DNA regions flanking the transcription start site (−5~+5 kb) were divided into 100 bins, each of 100 bp in size (Zhang and Li 2017). The binding signal of an HM in each bin was normalized by Eq. 3:

3 $ H_{l,m}^{\;i,j} = \frac{{n_{l,m}^{i,j} \times {{10}^9}}}{{{n_{l,m}} \times {l_i}}}, $

where $ H_{l,m}^{\;i,j} $ denotes the binding signal of the *l*-th HM in the *i*-th bin of the *j*-th gene within the *m*-th sample, $ n_{l,m}^{i,j} $ represents the read counts of the *l*-th HM mapped into the *i*-th bin of the *j*-th gene within the *m*-th sample. $ {n_{l,m}} $ describes the total read counts of the *l*-th HM in the *m*-th sample and *l*_*i*_ is the length of each bin.

### Extraction of key histone modifications

To estimate the influences of HMs on gene expression changes, HM signal changes were firstly defined as the ratio of average HM signals in tumor cells to that in normal cells and log2-transformed. The HM signal changes were then submitted to the RF algorithm to predict the up-DEGs from down-DEGs. For all DEGs, three-tenths of them were randomly selected as the training set, and the rest as the testing set. A 10-fold cross-validation method was adopted to test the prediction quality (Feng 2019). When all DEGs were scored, the average sensitivity (*S*_*n*_) and specificity (*S*_*p*_) were utilized to compute the area under the receiver operating characteristic curve (AUC), which was used to measure the effects of HM signal changes on gene expression changes.

4 $ 
 \left\{\begin{aligned} &
{S_n} = 1 - {{N_D^U} / {{N_U}}} \\&
 {S_p} = 1 - {{N_U^D} / {{N_D}}}
\end{aligned}\right.  , $

where ${N_D}$ and ${N_U}$ are the total number of down- and up-DEGs, respectively. $ N_D^U $ describes the number of up-DEGs that were incorrectly recognized as down-DEGs, $ N_U^D $ denotes the number of down-DEGs that were incorrectly recognized as up-DEGs.

### Identification of differential H3K79me2/H3K36me3 levels genes

Genes with differential H3K79me2/H3K36me3 levels between breast tumor and normal cell lines were quantified by Eq. 5. The genes with entropy < 1.549 were defined as genes with differential H3K79me2/H3K36me3 levels during cancerogenesis (Liu *et al.* 2013; Zhang *et al.* 2011).

5 $ 
 \left\{\begin{aligned} &
H_m^{\;j} = \sum\limits_{i \;= \;1} {H_m^{\;i,j}} ; SH_m^{\;j} = {{(H_m^{\;j} - {H_{m,\min }})} / {{H_{m,\max }}}} \\&
 {{P_m^{\;j} = SH_m^{\;j}} /{\sum\limits_{m\; = \;1} {SH_m^{\;j}} }}; {E_j} = - \sum\limits_{m \;=\; 1} {P_m^{\;j}{{\log }_2}} P_m^{\;j} 
\end{aligned}\right., $

where $ H_m^{\;i,j} $ describes the binding signal of H3K79me2/H3K36me3 in the *i*-th bin of the *j*-th gene within the *m*-th sample quantified by Eq. 3. $ {H_{m,\min }} $ and $ {H_{m,\max }} $ indicate the minimum and maximum signals of H3K79me2/H3K36me3 in the *m*-th sample. $ {SH}_{m}^{\;j} $ means standardized H3K79me2/H3K36me3 signal for the *j*-th gene within the *m*-th sample, and $ {E_j} $ is the Shannon entropy of H3K79me2/H3K36me3 for the *j*-th gene.

### Construction of risk scoring model

**Figure 6:**
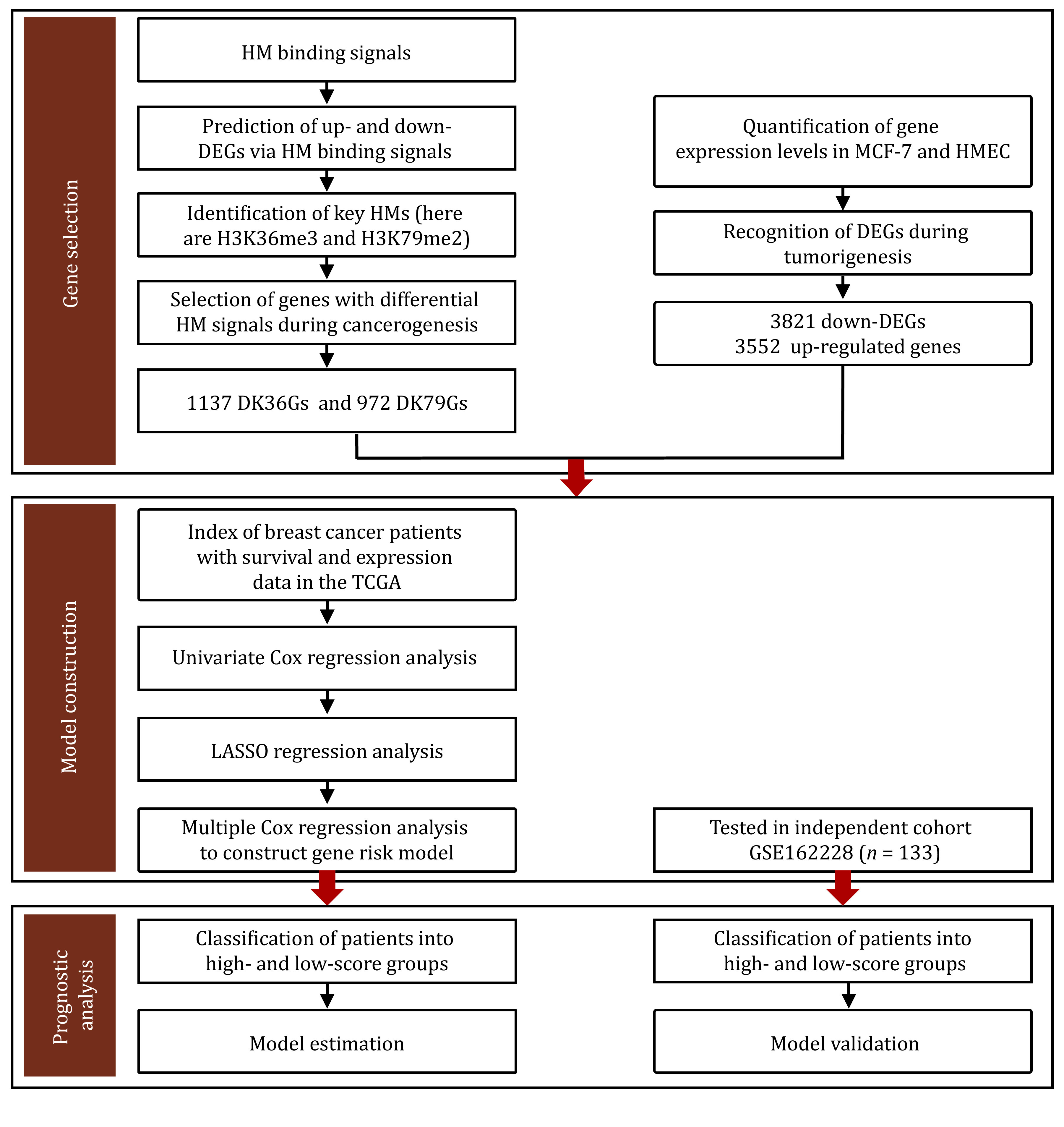
Computational framework. The DK79Gs or DK36Gs represent differential H3K79me2 or H3K36me3 level genes during cancerogenesis

By combining genes with differential H3K79me2/H3K36me3 levels during cancerogenesis, DEGs, RNA-Seq data and clinical profiles from the TCGA cohort, the clinical driver genes were identified as follows: (1) Univariate Cox regression was performed to assess the relationship between each candidate gene and survival time, genes with *P*-values < 0.05 were selected as seed genes. (2) LASSO and multivariate Cox regression were performed to select the driver genes from the seed genes, where the cut-off values were set as *P*-value < 0.05 and lambda. min = 5.51 × 10^−3^. On this basis, a risk score (RS) model comprising driver genes was constructed via Eq. 6:

6 $ RS = \sum\limits_{j\; =\; 1} {coe{f_j} \times {{\bar L}_{j,t}}} , $

here, *coef*_*j*_ is the regression coefficient calculated by multivariate cox analysis, and ${\bar L_{j,t}}$ represents the average expression level of the *j*-th gene in cancer samples. The computational framework was depicted in Fig. 6.

### Statistical analysis

Pathway analysis was carried out in the Hiplot software using GO enrichment analysis and KEGG pathway analysis (Yu *et al.* 2012). Survival curves were drawn via the Kaplan-Meier algorithm and conducted through the survfit function. Time-dependent receiver-operating characteristic (ROC) curves were used to assess model performance and plotted by the survivalROC function. Univariate and multivariate Cox analyses were achieved through the ‘coxph’ function. LASSO regression was implemented by the cv.glmnet function. The percent increase in the mean square error (IncMSE) for each parameter in the RF algorithm was calculated and transformed into a rank value to describe its contribution to gene expression changes. And parameters with lower rank values represent higher IncMSE values and greater contributions to gene expression changes.

## Conflict of interest

Ling-Yu Wang, Lu-Qiang Zhang, Qian-Zhong Li and Hui Bai declare that they have no conflict of interest.
